# Overview of Chagas disease surveillance in an endemic region in Southeastern Brazil

**DOI:** 10.1590/S1678-9946202365051

**Published:** 2023-10-09

**Authors:** Aline Ferreira Rafael, Raquel Aparecida Ferreira, Ariela Ferreira Mota, Renata Fiúza Damasceno, Agna Soares da Silva Menezes, Bartolomeu Teixeira Lopes, Gustavo Libério de Paulo, Ester Cerdeira Sabino, Antônio Luiz Pinho Ribeiro, Nayara Dornela Quintino, Thallyta Maria Vieira

**Affiliations:** 1 Universidade Estadual de Montes Claros, Programa de Pós-Graduação em Ciências da Saúde, Montes Claros, Minas Gerais, Brazil; 2 Fundação Oswaldo Cruz, Instituto René Rachou, Grupo de Pesquisa Triatomíneos, Belo Horizonte, Minas Gerais, Brazil; 3 Superintendência Regional de Saúde de Montes Claros, Minas Gerais, Brazil; 4 Universidade Federal de Minas Gerais, Instituto de Geociências, Programa de Pós-Graduação em Análise e Modelagem de Sistemas Ambientais, Belo Horizonte, Minas Gerais, Brazil; 5 Universidade de São Paulo, Faculdade de Medicina, Instituto de Medicina Tropical de São Paulo, São Paulo, São Paulo, Brazil; 6 Universidade Federal de Minas Gerais, Faculdade de Medicina, Hospital das Clínicas Belo Horizonte, Minas Gerais, Brazil; 7 Superintendência Regional de Saúde de Divinópolis, Minas Gerais, Brazil

**Keywords:** Chagas disease, Health Surveillance, Control, Neglected diseases

## Abstract

Chagas disease (CD) is a neglected disease caused by the protozoan *Trypanosoma cruzi*. It has high morbidity and mortality rates and mainly affects socially vulnerable populations. This is a cross-sectional study, with retrospective and prospective data collection. Using questionnaires applied to environmental surveillance coordinators, we characterized the status of CD surveillance activities in municipalities endemic for the disease in Northern Minas Gerais State (MG) and Jequitinhonha Valley (Vale do Jequitinhonha). Moreover, we spatialized the vulnerability index for chronic CD in the study area. The population consisted of 22 environmental surveillance coordinators, active in 2020, from Northern MG and Jequitinhonha Valley, 21 municipalities included in the SaMi-Trop research project, and Montes Claros municipality. After applying the questionnaires to the coordinators, a descriptive analysis of the variables was performed. To characterize the active municipalities, the explanatory variables collected in the questionnaire were compared with the dichotomous variable. Bivariate descriptive analysis was performed. Finally, geoprocessing techniques were used to spatialize the data and prepare maps. Regarding the team of endemic combat agents (ECA), 90.9% reported the lack of a specific team for CD vector control actions. Of the 22 municipalities participating in this study, nine were active (41.1%). Only 25% (n=2) of active municipalities (9% of the municipalities studied) met the target of visiting 50% of households per year. Finally, 81.1% of the coordinators stated that in their municipality, they developed actions linked to primary health care (PHC). The implementation of CD surveillance activities weakened in the endemic region. Few municipalities have a surveillance team, with low regularity of active surveillance and noncompliance with the program’s goal. The results suggest insufficient recording of activities in the information system, considering that there are municipalities that report performing the activities, but no production record was observed in the system.

## INTRODUCTION

Chagas disease (CD) is a neglected disease caused by the protozoan *Trypanosoma cruzi* ^1^. The disease has high morbidity and mortality rates^2^ and can lead to a high financial cost to health services^3^. It mainly affects socially vulnerable populations, with difficulties in accessing health services and low perception of health quality^4-6^.

The last comprehensive serological survey conducted in Brazil in the early 1980s showed a prevalence of 8.83% of CD cases in Minas Gerais State (MG), the second highest seropositivity rate in Brazil^7^. The state also recorded the highest number of deaths (n=12,902) from the disease from 2007 to 2017, and the Jequitinhonha (0.593) and Northern MG (0.550) macro-regions had the highest vulnerability index values for chronic CD. This index shows the areas with the highest risk of morbidity and mortality in chronic CD^8^.

The first diagnosed case of CD in the world was in 1909, in the Lassance municipality^1^. However, a nationwide disease surveillance program only began to be developed in Brazil in 1976, known as the Chagas disease control program (Programa de Controle da Doenca de Chagas [PCDCh]), implemented and conducted under the responsibility of the Superintendence of Public Health Campaigns (Superintendencia de Campanhas de Saude Publica [SUCAM]). Although the PCDCh was very successful throughout Brazil, at the end of the 1990s, the process of decentralization of program^9^ actions began, so that responsibility for organizing and developing entomological surveillance activities was transferred to the states and municipalities, under the coordination of the regional health units (RHU)^10,11^.

Since decentralization, CD vector control activities have lost priority and are not recognized as a program, but as an isolated action. Among the activities performed, the following stand out: active surveillance, passive surveillance (notification of the presence of triatomines in households by the population, called popular participation), taxonomic classification and evaluation of triatomines by entomology laboratories, educational actions, updating of geographic recognition, and chemical control, when necessary^11-13^ Regarding active surveillance, the recommendation is that in high-risk areas, such as Northern MG and Jequitinhonha, the municipality’s endemic disease control agent should be responsible for investigating the occurrence of triatomines in 50% of the localities annually^14^. From the management point of view, the coordination of CD vector control actions is the responsibility of the environmental surveillance coordinator. They are responsible for managing the surveillance, prevention, and control of zoonoses and vector-borne diseases at the municipal level, which makes them the primary professional in vector control^15^.

Along with the unsuccessful decentralization process, the eradication of the main vector of CD was announced in 2006. As a result, a false idea of eradicating the disease was installed in Brazil, due to the success in controlling the vector species *Triatoma infestans* (Klug, 1834)^16^. Brazil has about 65 native species of triatomines with different vectoring abilities^17^. In this context, with the disarticulation of surveillance actions, the definition of priority areas for intervention and care related to CD was limited, since control actions and chronic cases of the disease are currently not well known^18^. The result is that many municipalities do not prioritize vector control activities and epidemiological surveillance of CD, both acute and chronic^11,19^. In MG, as a strategy to give visibility to the surveillance of the chronic form of the disease, chronic CD became a compulsory notification in 2018^20^. Chronic CD was included in the Brazilian list of notifiable diseases of compulsory notification only in 2020^13^.

Therefore, this study aimed to characterize, using questionnaires applied to environmental surveillance coordinators, the status of CD surveillance activities in municipalities endemic to the disease in Northern MG and Jequitinhonha Valley. Additionally, the spatialization of the vulnerability index for chronic CD was performed in the study area.

## MATERIALS AND METHODS

This is a cross-sectional study, with retrospective and prospective data collection

### Study area

**Figure 1:**
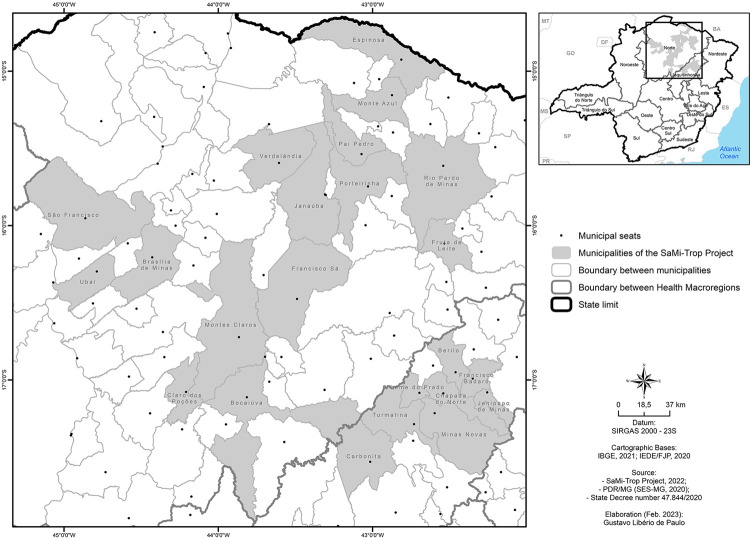
Location of the study area: municipalities in northern Minas Gerais and Jequitinhonha Valley, endemic regions for Chagas disease, 2020.

The study was conducted in Northern MG and Jequitinhonha Valley, including the 21 municipalities that are part of the Sao Paulo-Minas Gerais Tropical Medicine Research Center (SaMi-Trop) project. Montes Claros municipality was added as it is the seat of the health macro-region of Northern MG. Figure 1 shows the location of the study area, with the 22 participating municipalities. The study area is located between the geographical coordinates 42°4’45.01”–45°15’27.96”W and 14°38’59.41”–17°55’31.44”S.

SaMi-Trop consists of a network of collaborating scientists from Minas Gerais and Sao Paulo States, which was created with the purpose of conducting research projects to find new biomarkers for CD and validate existing ones. It presents a cohort of patients with CD in these 21 municipalities in Northern MG and Jequitinhonha Valley, who were selected due to a high prevalence of chronic CD, based on patient self-report from the Telessaude database^21,22^.

## Study population and data collection

### Characterization of the status of Chagas disease surveillance activities

The study population included environmental surveillance coordinators from the 22 municipalities, active in October 2020. For data collection, the questionnaire adapted from Villela *et al*.^10^ was used. It was adapted by five specialists from the Minas Gerais State Health Superintendence who worked on the subject of CD. These professionals analyzed the content of the questions and the possible answers, making suggestions and criticisms, and assessed the relevance and clarity of each item (alternating with yes/no answers). The tool was reformulated and the questions considered relevant by at least four of the specialists were kept. Finally, the questionnaires were sent by WhatsApp to the coordinators of the 22 municipalities, with a deadline of 30 days to return them to the researchers.

The questionnaire consisted of 42 questions and was structured in three blocks:

- Profile of professionals working in CD control activities;
- Status of CD surveillance activities according to active municipalities;
- Difficulties and limitations for the implementation of activities.

During completion, each participant read the questions of the questionnaire and, according to their perception/knowledge, answered yes or no (for 13 questions). For the 29 other questions, participants chose the alternative that they considered correct or more pertinent.

#### Characterization of active municipalities and characterization of their rural and urban areas

For the description of active municipalities, the existence or not of vector control actions in the municipalities was observed. For the construction of these variables, the production reports of passive and active surveillance activities performed from 2018 to 2020, extracted from the Chagas Disease Control Program Information System (Sistema de Informacao do Programa de Controle da Doenca de Chagas [SISPCDCh]) were considered. Municipalities with at least one activity recorded in at least two years were considered active.

#### Characterization of rural and urban areas and spatialization of the study area according to IBGE data (2017)

To characterize the spatialization of the municipalities, the IBGE classification of rural and urban areas was used. For this classification, three criteria were considered: demographic density, location in relation to the main urban centers, and population size. After analyzing the criteria, the municipalities were characterized as “urban,” “rural,” or “intermediate.” They are further subclassified as remote or adjacent. Municipalities that are relatively close to the national average are considered remote in relation to the major hierarchies of the nearest REGIC (Areas of Influence of Cities: metropolis, regional capital, subregional center). Adjacent areas are those whose distance is equal to or less than the national average in relation to at least one of the REGIC centers^23^.

#### Spatialization of vulnerability indices for chronic Chagas disease in areas with or without vector control actions

For the spatialization of the areas of vulnerability for chronic CD, public data made available by the Department of Health Surveillance of the Brazilian Ministry of Health (SVS/MS) in a 2022 epidemiological bulletin^8^ were used.

## Data analysis

To characterize the status of CD surveillance activities in the municipalities, descriptive analysis of the variables was performed, estimating absolute (n) and relative (%) frequencies. For active municipalities, the explanatory variables collected in the questionnaire were compared with the dichotomous variable (active municipality – yes or no). Bivariate descriptive analysis was performed. All analyses were performed in the Statistical Package for the Social Sciences (version 19, SPSS Inc., IBM, Armonk, New York, USA) statistical software.

Geoprocessing techniques were used to spatialize the data and prepare maps, using the following software: Google Earth, for exploratory visualization; ArcGIS 10.8 and QGIS 3.28 (Open Source Geospatial Foundation, Chicago, USA), for data processing, spatial analysis, and cartographic representation (within cartographic precepts); and Microsoft Office Excel, for structuring databases, automated calculations, and elaborating graphic representations.

## Ethical aspects

This study was approved by the Research Ethics Committee of the State University of Montes Claros (CAAE 33418720.2.0000.5146), in compliance with Resolution 466/2012 of the National Health Council^24^. All participants signed an informed consent form.

## RESULTS

### Characterization of the status of Chagas disease surveillance activities and active municipalities

All 22 municipal environmental surveillance coordinators answered the questionnaire. Of this total, most were men (68.2%) with a mean age of 39 years (Table 1). The academic profile shows that 45.5% completed higher education and 54.5% completed secondary education (Table 1). Considering their link with the municipal health service, 54.5% had temporary contracts and most of them (59.1%) had been a coordinator for more than five years. Regarding the team of endemic combat agents (ECAs), 90.9% reported the lack of a specific team for CD vector control actions and 86.3% of the contractual team worked in the position for up to four years. In contrast, 59.1% of the staff have been in office for more than five years. Only 54.5% of the coordinators developed actions in partnership with primary health care (PHC) (Table 1).

**Table 1:** Profile of professionals working in Chagas disease control activities (n=22).

Characteristic	Descriptive
**Characteristics of the endemic coordinators**	***n (%)***
Age, mean (SD)	39 (10.2)
Sex	
Men	15 (68.2%)
Women	7 (31.8%)
Schooling level	
Complete secondary education	12 (54.5%)
Complete higher education	10 (45.5%)
Link to the Health Department	
Hired	12 (54.5%)
Hired via selection process	10 (45.5%)
Time in the position	
Up to two years	4 (18.2%)
Two years	5 (22.7%)
More than five years	13 (59.1%)
**Characteristics to combat endemic diseases**	
Existence of specific professionals for PCDCh	
No	20 (90.9%)
Yes	2 (9.1%)
Length of service of the contractual team	
Up to four years	19 (86.3%)
More than five years	3 (13.6%)
Length of service of the effected team	
Up to four years	9 (40.9%)
More than five years	13 (59.1%)
Partnership between PHC and PCDCh	
No	6 (27.3%)
Yes	12 (54.5%)
Information not available	4 (18.2%)

PHC = primary health care; PCDCh = Chagas disease control program.

Regarding the activities recommended by the program, 90.9% of the 22 municipalities performed passive surveillance; 72.7% performed active surveillance and laboratory surveillance; 90.9% performed chemical control, when necessary; and 77.3% promoted environmental education (Table 2). Most environmental surveillance coordinators stated that ECAs always receive the triatomine laboratory evaluation report and 72.7% reported that the population always receives feedback on the triatomine laboratory evaluation.

**Table 2:** Status of Chagas disease surveillance activities according to active municipalities (n=22).

Characteristics	Descriptive n (%)	Bivariate	

		Active municipality	

		Yes n (%)	No n (%)
The municipality met the goal			
No	14 (63.6%)	7 (50%)	7 (50%)
Yes	8 (36.4%)	2 (25%)	6 (75%)
Date the goal was last met			
2007	1 (4.5%)	1 (100%)	0 (0%)
2012	1 (4.5%)	0 (0%)	1 (100%)
2015	5 (22.7%)	3 (60%)	2 (40%)
2016	1 (4.5%)	1 (100%)	0 (0%)
2017	3 (13.6%)	0 (0%)	3 (100%)
2018	2 (9.1%)	0 (0%)	2 (100%)
2019	3 (13.6%)	1 (33.3%)	2 (66.7%)
2020	1 (4.5%)	1 (100%)	0 (0%)
Information not available	5 (22.7%)	2 (40%)	3 (60%)
**Vector control activities performed in the municipality**			
Passive surveillance			
No	2 (9.1%)	0 (0%)	2 (100%)
Yes	20 (90.9%)	9 (45%)	11 (55%)
Active surveillance			
No	6 (27.3%)	2 (33.3%)	4 (66.7%)
Yes	16 (72.7%)	7 (43.8%)	9 (56.2%)
Laboratory surveillance			
No	6 (27.3%)	2 (33.3%)	4 (66.7%)
Yes	16 (72.7%)	7 (43.8%)	9 (56.2%)
Chemical control			
No	2 (9.1%)	0 (0%)	2 (100%)
Yes	20 (90.9%)	9 (45%)	11 (55%)
Environmental education			
No	5 (22.7%)	2 (40%)	3 (60%)
Yes	17 (77.3%)	7 (41.2%)	10 (58.8%)
The population is guided on management			
No	4 (18.2%)	1 (25%)	3 (75%)
Yes	18 (81.8%)	8 (44.4%)	10 (55.6%)
The population receives results on recovered triatomines			
Sometimes	4 (18.2%)	2 (50%)	2 (50%)
Always	16 (72.7%)	7 (43.8%)	9 (56.2%)
Information not available	2 (9.1%)	0 (0%)	2 (100%)
ECAs receive the triatomine test results from the searches they participate in			
Sometimes	2 (9.1%)	0 (0%)	2 (100%)
Always	20 (90.9%)	9 (45%)	11 (55%)
**Difficulties and limitations in implementing activities**			
Specific team to receive triatomines			
No	13 (59.1%)	7 (53.8%)	6 (46.2%)
Yes	9 (40.9%)	2 (22.2%)	7 (77.8%)
Trained professionals to identify and test triatomines*			
No	15 (68.2%)	5 (33.3%)	10 (66.7%)
Yes	6 (27.3%)	3 (50%)	3 (50%)
Material resource for laboratory analysis*			
No	13 (59.1%)	6 (46.2%)	7 (53.8%)
Yes	8 (36.4%)	2 (25%)	6 (46.2%)
**Difficulties and limitations in implementing activities**			
Lack of PITs			
No	17 (77.3%)	7 (41.2%)	10 (58.8%)
Yes	4 (18.2%)	1 (25%)	3 (75%)
Lack of training and updating*			
No	10 (45.5%)	4 (40%)	6 (60%)
Yes	11 (50%)	4 (36.4%)	7 (36.6%)
Link to PHC*			
No	18 (81.1%)	8 (44.4%)	10 (55.6%)
Yes	3 (13.6%)	0 0%)	3 (23.1%)

^*^refers to municipalities without an answer.

Of the 22 municipalities participating in this study, nine were active (41.1%) (Table 2). Comparing the status of CD surveillance activities among the municipalities, only 25% (n=2) of active municipalities (9% of the municipalities studied) met the target of visiting 50% of households per year—some municipalities had not met the goal since 2007. About 55% of the municipalities were considered inactive, that is, they had no record of vector surveillance activities in the information system (SISPCDCh), but reported performing activities recommended by the program.

Regarding the difficulties and limitations in implementing CD vector control activities, 40.9% of the coordinators stated the lack of a specific professional to receive triatomines in their municipalities and 36.4% had limitations in terms of material resources for laboratory analysis. In total, 27.3% of the municipalities did not have a professional trained to identify or examine triatomines. However, a minority (18.2%) did not have a triatomine information office (Posto de Informacao de Triatomineo [PIT]) in their municipality. Finally, 81.1% of the coordinators performed actions in association with the PHC in their municipality. The profile of active municipalities showed that only 22% had a specific team to receive triatomines, in contrast to 77.8% of inactive municipalities. On the other hand, 50% of the municipalities had a professional trained to identify and/or examine triatomines, regardless of whether they were active or inactive.

**Figure 2:**
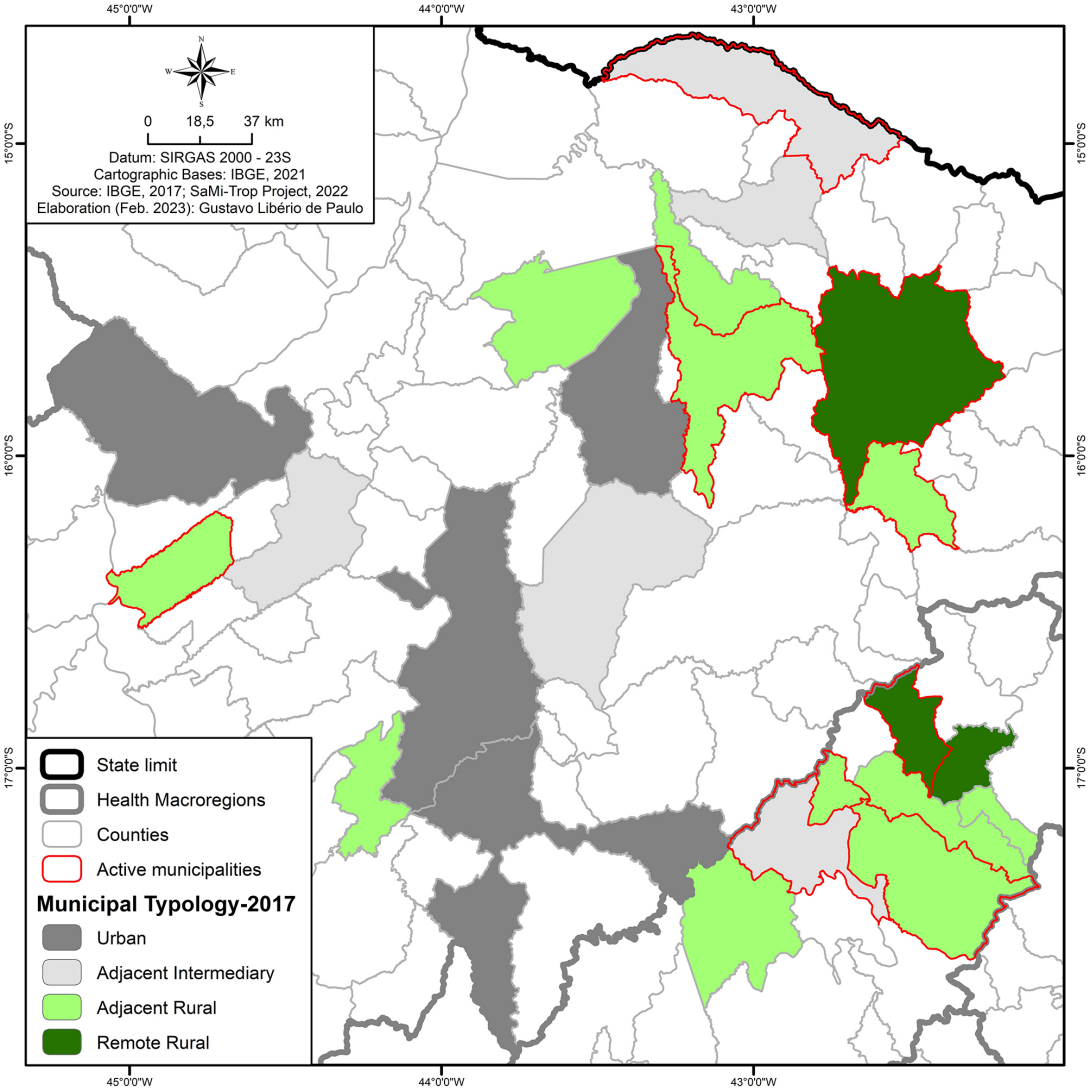
Classification map of rural-urban municipality typologies23.

Figure 2 shows that among the 22 participating municipalities, 11 were classified as adjacent rural, of which five actively promoted vector control actions. Moreover, three municipalities were classified as remote rural, and two of them performed vector control activities. Finally, among the five municipalities classified as adjacent intermediate, only two recorded vector control activities (Figure 2). No municipality classified as urban had records of vector actions during the period observed.

**Figure 3:**
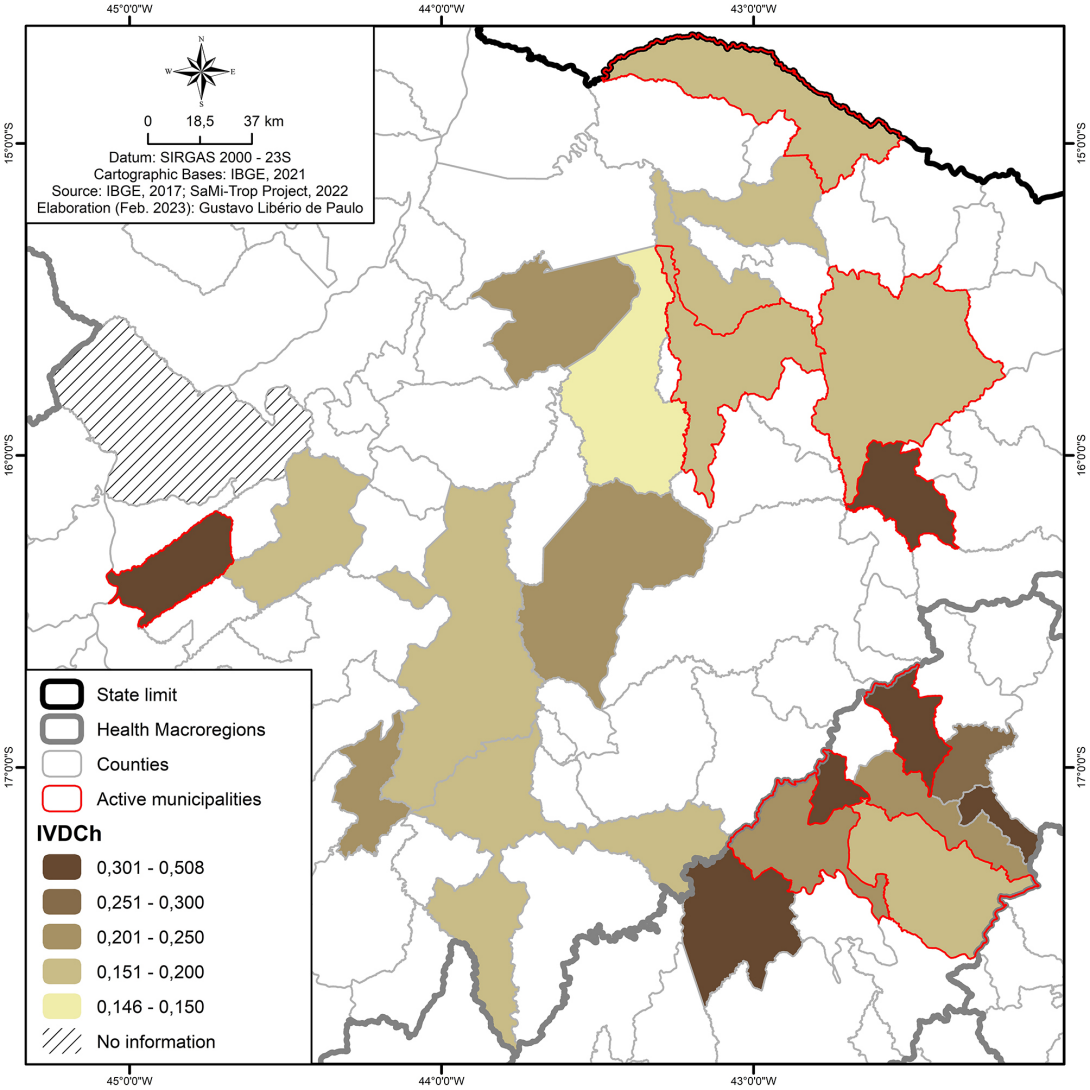
Vulnerability index map for chronic Chagas disease8.

Figure 3 provides a spatial visualization of the vulnerability indices for chronic CD in areas that did or did not perform control actions at the time of the study. On a scale of 0 to 1, the closer the territory is to 1, the more vulnerable it is to chronic CD. In our findings, four municipalities with a high vulnerability index for CD promoted vector control actions.

## DISCUSSION

This study showed that CD surveillance and vector control actions were poor in municipalities in Northern MG and Jequitinhonha Valley, as only nine municipalities registered their activities in SISPCDCh. This is a concerning scenario, considering that the study site refers to municipalities with a high prevalence of chronic CD, identified as part of the SaMi-Trop^22^ cohort. Moreover, these areas correspond to the regions of Brazil with high priority for intervention and care related to chronic CD^25^. This negligence is probably a direct consequence of the fragmentation of the program resulting from the process of decentralization of vector control activities^19,26^.

The organization of surveillance activities in the municipal context depends on a minimum team, with a coordinator, a data recorder, and ECAs^27^. In a scenario observed in the region, most environmental surveillance coordinators are temporary contracts. Low investment can lead to high employee turnover, a fact that has already been correlated in other professional categories in previous studies^10,28^. The failure to structure the team may be partly related to the invisibility of the disease and the failure to prioritize triatomine control, to the detriment of other endemic diseases^17^. Another factor is the technical and operational loss due to decentralization, since many Brazilian municipalities do not have ECAs to perform CD vector control activities. In this study, among the municipalities that have these professionals, most are temporary contracts, which hinders the sustainability of actions in the municipal context^10^. Moreover, many professionals from the now-defunct SUCAM have retired or are in the process of retiring, thus withdrawing their technical and managerial support to the municipalities and interrupting field supervision and continuing education actions, which hinders the transfer of knowledge and the continuity of the program^29,30^. Regarding the profile of ECAs, most municipalities did not have a specific team to perform vector control actions. One aspect that apparently aggravates the formation and prioritization of a CD technical team is the lack of notification of CD cases in the territories. In a study conducted in municipalities in another endemic region in MG, the authors state that in the absence of reported cases of CD, other endemic diseases with a higher number of reported cases in the area, such as dengue and Zika^31^, receive budgetary and political prioritization. Moreover, our findings showed that some municipalities performed vector control actions, but the information system presented no production record. This reinforces invisibility and loss of priority on local schedules.

Most municipalities reported integration between surveillance and PHC. Even with this finding, we reinforce the importance of greater communication between ECAs and other PHC professionals, thus strengthening the better screening of patients with an epidemiological history of CD, and notification of possible patients identified by ECAs in active or passive surveillance^32^. Most of the municipalities with links to PHC have no record of entomological control activities. From this perspective, this gap contributes to the invisibility and permanence of the disease in endemic and non-endemic regions^33^.

Regarding the entomological control actions promoted by the municipalities, a significant number did not produce management reports on the activities performed in the period observed. According to the coordinators’ report on meeting the goal of active surveillance provided by the Minas Gerais State Health Department (SES/MG), most municipalities did not visit 50% of their households every year. Of the eight municipalities that were up to date with this activity, only two were really active, according to the production report. However, one municipality reported having achieved this goal more than 10 years ago. This result suggests, on the one hand, a possible lack of actions in the municipalities and, on the other hand, a great weakness in the recording of activities in the official information system (SISPCDCh). PCDCh, the information system currently used to record entomological surveillance activities, has a structural gap. However, although it is an obsolete technology, this does not justify the lack of recording of activities in the official system.

Another important activity in the context of the program is passive surveillance. For most coordinators, this was not a problem in their municipality, as most municipalities have PITs installed. In contrast, they reported the lack of a specific person to receive triatomines. Therefore, the physical existence of PITs without the presence of triatomine receivers may point to passive surveillance, which, in fact, is not effective in the municipalities. The disarticulation between services and their flow as a result of the process of decentralization of health caused the control strategies to be adrift, poorly established, and disseminated between municipalities and the population. Other studies have been pointing the lack of knowledge of the population about triatomines and PITs^34,35^.

Of the 22 municipalities participating in this study, 14 are located in rural areas. The program’s methodology is directed to rural areas^11^, as our findings confirm, since no active municipality has urban characteristics. However, although CD has been known as a predominantly rural disease, studies show the occurrence of triatomines in urban areas, a change resulting from alterations in the epidemiological eco-profile of triatomines^36^. This reinforces the need to strengthen surveillance in rural and critical urban areas.

Regarding vulnerability, four municipalities active in entomological control actions had a high index. In a positive way, they seemed to be concerned and, to a certain extent, prioritize the promotion of activities compared with other municipalities. However, more in-depth analyses show that there are few municipalities in which this prioritization appears this issue, reinforcing the profile of a highly neglected disease. Moreover, CD is no longer limited to endemic areas, but is present in urban areas and non-endemic countries due to migratory movements. Therefore, it is no longer limited to neglected populations either. However, it is still not treated with the necessary priority, given the millions of people affected by the disease or at risk of infection.

The great issue of neglected diseases is that they are characterized as being restricted to populations in unfavorable socioeconomic situations and with low schooling levels. This profile extends to precarious housing conditions, poor health education, and little access to health systems. Thus, CD is a global public health problem that is isolated in regions of poverty and scarcity and is not treated as a priority^37^.

Even with important results that can help strengthen the CD surveillance program in Northern MG and Jequitinhonha Valley, this study has limitations. The main limitation is related to the number of participating municipalities, which hindered the assessment of factors associated with the absence of activities in the program. In contrast, the selected municipalities are among the 21 with the highest prevalence of CD in MG, according to a large database of the Minas Gerais teleassistance network^22^.

## CONCLUSION

The implementation of CD surveillance activities in the study municipalities, located in endemic regions and classified as high risk, weakened. Few municipalities have a specific team for CD, with a non-regularity of active surveillance resulting in the non-compliance of the program goal. The results suggest insufficient recording of activities in the information system (SISPCDCh), considering that some municipalities reported performing the activities, but did not recorded them in the information system.

This scenario is worrying, as MG is a high-priority Brazilian region, with a high prevalence of CD, and the Northern MG and Jequitinhonha Valley have the highest indicators of vulnerability to chronic CD in Brazil. The results of this study can support the development of public policies to strengthen CD surveillance, especially vector control actions.
